# Endoscopic Surgery Without Decompressive Craniectomy in Large Putaminal Intracerebral Hemorrhage: Assessment of Efficacy and Safety

**DOI:** 10.1007/s12028-019-00880-8

**Published:** 2019-12-16

**Authors:** Yuanliang Ye, Qiujing Wang, Weiyang Ou, Jian He, Zhenhui Zhao

**Affiliations:** 1 The National Key Clinical Specialty, The Engineering Technology Research Center of Ministry of Education of China, Guangdong Provincial Key Laboratory on Brain Function Repair and Regeneration, Department of Neurosurgery, Zhujiang Hospital, Southern Medical University, Guangzhou, 510282 Guangdong Province China; 2 Department of Neurosurgery, Liuzhou General Hospital, Liuzhou, 545001 Guangxi Autonomous Region China

**Keywords:** Endoscopic surgery, Intracerebral hemorrhage, Outcome, Cerebrospinal fluid drainage

## Abstract

**Background:**

Decompressive craniectomy (DC) is performed conventionally for large putaminal intracerebral hemorrhage (ICH). However, DC causes local skull defect and leads to post-surgical cranioplasty. The aim of this study is to investigate the effectiveness and safety of an endoscopic procedure to treat large putaminal ICH without DC.

**Methods:**

This retrospective study included 112 large putaminal ICH patients who underwent hematoma evacuations with either an endoscopic procedure (group A) or with DC (group B) between January 2009 and June 2017. The efficacy was evaluated by mean modified Rankin Scale (mRS) three months after surgery. Safety was evaluated by mortality rate and postoperative complications. Univariate and multivariate logistic regression analyses were performed to determine the risk factors for clinical outcomes.

**Results:**

The study included 49 patients in group A and 63 in group B. The mRS scores in both groups were similar after 3 months’ follow-up (*p* = 0.709). There was no difference in the mortality rate between the two groups (*p* = 0.538). The rate of complications was lower in group A than that in group B (*p* = 0.024). Smaller preoperative midline shift (*p* = 0.008) and absent intraventricular extension (*p* = 0.044) have contributed significantly to better outcomes.

**Conclusion:**

Endoscopic hematoma evacuation without DC is safe and effective for patients with large putaminal ICH and deserves further investigation, preferably in a randomized controlled setting.

## Introduction

Spontaneous intracerebral hemorrhage (ICH) is one of the most deadly forms of stroke with an extremely high mortality rate [1, 2]. Patients with large spontaneous ICH experience poor outcomes [3–7]. Although the International Surgical Trial in Intracerebral Hemorrhage study showed that emergency surgical hematoma evacuation failed to improve the outcome of ICH patient comparing with the same type of patients who received initial medical management without surgery [8], surgical treatment of large ICH has been considered as a life-saving intervention. Currently, the prompt measure to reduce an increasing intracranial pressure (ICP) is primarily a surgical approach through either only decompressive craniectomy (DC) or DC with hematoma evacuation. However, DC could bring many delayed complications and might eventually lead to cranioplasty after surgery.

With the rapid advancement of imaging software and transparent tube sheath, endoscopic surgery has gradually become an effective and well-accepted method for treating ICH [9–13]. Endoscopic hematoma evacuation has become well received for its high evacuation rate and less complication, especially in patients with moderate-sized hematoma [14]. In the present study, we applied endoscopic surgery without DC for patients with large putaminal ICH and assessed the efficacy and safety of this procedure. We also investigated the impact of preoperative clinical factors on the surgical outcomes.

## Methods

### Patients

Between February 2014 and June 2017, 49 patient with large putaminal ICH received the endoscopic procedure (group A). The group A patients were matched to a group of 63 patients who were treated, between January 2009 and June 2014, with DC along with hematoma evacuation (group B). The intensive care unit, medical, and operating room practices had been kept the same during both study periods. The patients and their family members consented to accept serious neurologic deficits as a consequence of the procedures. The ICP was monitored with a cannula placed in the contralateral lateral ventricle at the end of surgery. The study was approved by the ethics committee of Zhujiang Hospital in agreement with the Declaration of Helsinki. All research was performed in accordance with relevant guidelines and regulations. Informed consent was obtained by the ethics committees. The family members signed the written surgical consent before surgery.

### Inclusion and Exclusion Criteria

Patients were included or excluded in the study according to the following criteria. Inclusion criteria: (1) spontaneous ICH in putamen based on computed tomography (CT) scan; (2) the volume of the hematoma was greater than 50 ml; (3) the patients’ family consented and signed the proxy for the procedure. Exclusion criteria: (1) the ICH was caused by trauma, tumor, moyamoya, aneurysm, or vascular malformation; (2) the patient was accompanied by severe cardiac, hepatic, renal, or pulmonary dysfunctions; (3) patients did not have a follow-up CT scan 7 days after surgery; (4) patient’s family or legal representative did not consent to surgical treatment.

### Surgical Procedures

**Fig. 1:**
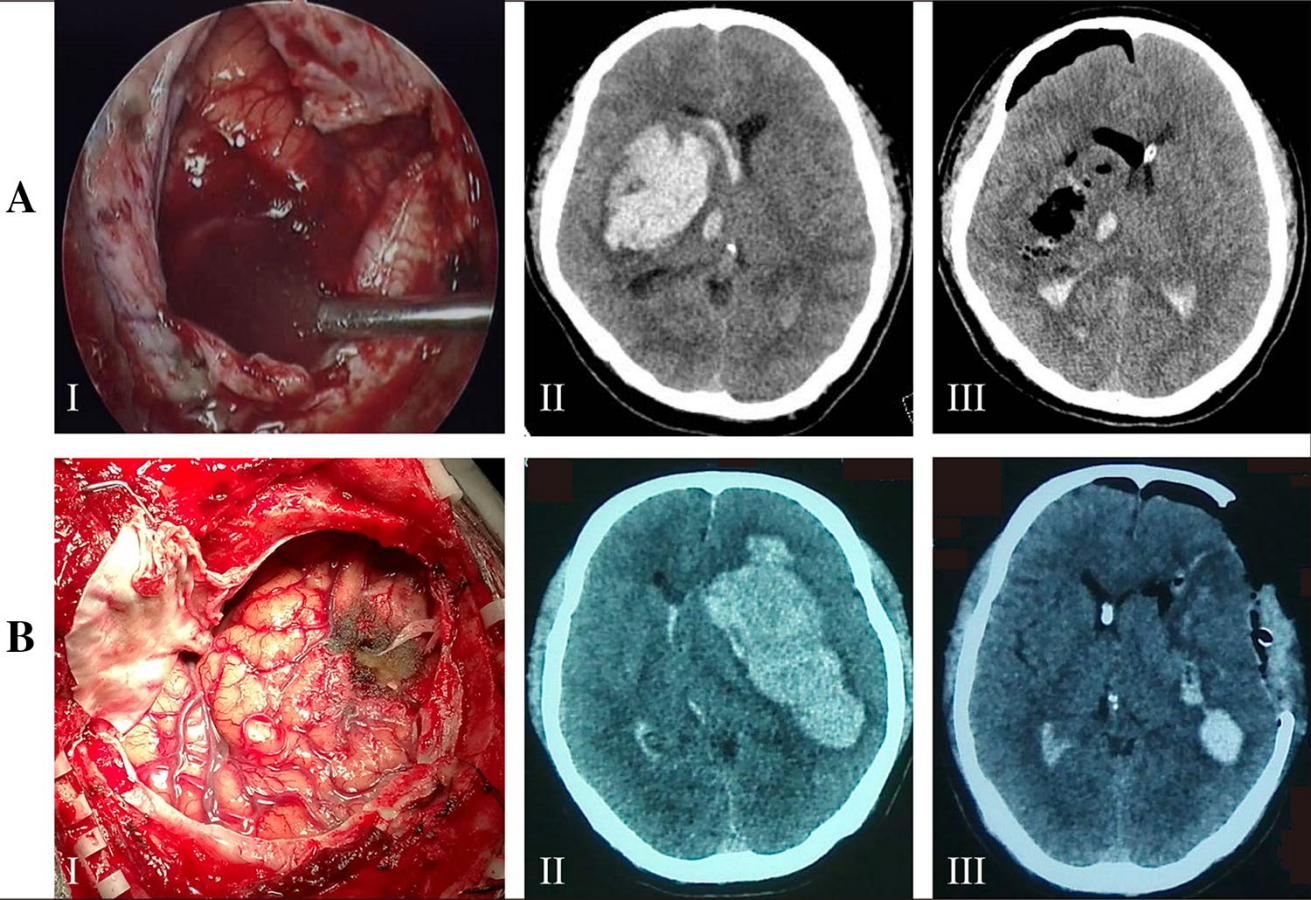
**a** Endoscopic surgery for large putaminal ICH without DC. The brain tissue collapsed immediately after endoscopic hematoma evacuation (I). Preoperative CT shows right hemispheric large ICH (II), and postoperative CT shows well-evacuated ICH with slight edema (III). **b** DC with hematoma evacuation for large putaminal ICH. A large frontoparietal craniectomy is performed, and hematoma is evacuated by endoscope (I). Preoperative CT shows left hemispheric large ICH (II), and postoperative CT shows the satisfactorily evacuated ICH, large left frontoparietal craniectomy, and a cannula in right ventricles (III)

In group A, a 4-cm skin incision was made on the frontal or temporal scalp of hemispheric ICH. The entry site of craniotomy was widened to 20 to 25 mm in diameter. Dura mater was incised and opened, and a small size incision on the cortical pia was performed. A transparent plastic sheath was inserted, with its insertion distance based on the preoperative CT scan, to the core of the hematoma. The rigid endoscope measuring 0° and 4 mm in diameter (Karl Storz, Germany) was held in the left hand, while a suction cannula was performed in the right hand. Once the sheath was steady, the rigid endoscope and suction cannula were then introduced into the hematoma cavity through the endoscopic corridor. The hematoma was removed while the sheath was gradually withdrawn. The cerebral cortex would collapse as long as the hematoma was removed satisfactorily (Fig. 1a:I–III). For patients in group B, surgery was performed by removing a large bone flap, and then hematoma was removed via endoscope or under microscope. The size of DC was approximately 10–11 cm (anterior–posterior) by 8–9 cm (from temporal bone base to superior sagittal sinus). Duraplasty was implemented by using galea-periost flap (Fig. 1b: I–III). ICP was monitored through a cannula placed in the contralateral lateral ventricle at the end of surgery.

### Postoperative Management

Assessment of Glasgow Coma Scale (GCS) was executed every 2 h for 7 days after surgery. Brain CT scan was performed immediately after surgery and repeated every day for 7 days postoperatively. However, if the patient showed signs of deterioration, immediate brain CT scan was carried out. A lumbar catheter drain was applied after surgery in order to observe the clarification of the cerebrospinal fluid (CSF). In case of infection, patients would receive amikacin (20 mg, 1–2 times/day) introduced into the vertebral canal. Postoperative analgesic sedation was carried out during 24 h after surgery. Conservative management included mannitol, glycemic control, and intensive blood pressure management. Tracheotomy was used when poor capacity of expectoration occurred. ICP monitoring was undertaken routinely in this study.

#### Clinical and Radiological Follow-Up

Complications, GCS, mortality, and modified Rankin Scale (mRS) were documented for 3 months after surgery. Meningitis was considered present with symptoms such as positive meningeal irritation sign, CSF leukocyte count > 1000/mm^3^, and positive CSF bacterial culture. Postoperative hydrocephalus, extra-axial fluid collection, and cerebral infarction were identified on postoperative CT scans. The mRS scores were defined as favorable (mRS 0–3) and unfavorable (mRS 4–6). Pre- and postoperative hematoma volumes were assessed by Slicer software, and the rate of hematoma evacuation was calculated as follows: Preoperative hematoma volume–postoperative hematoma volume]/preoperative hematoma volume × 100%. Brain edema volumes at 7 days postoperative were measured using semi-automatic volumetry, with a threshold-based algorithm (5–33 HU).

### Statistical Analysis

All statistical analyses were performed using *SPSS* 22 for Windows (*SPSS* Inc., Chicago, Illinois). Continuous variables were expressed as mean ± standard deviation or median with interquartile range depending on the distribution of the variables. Categorical variables were expressed in frequency or as a percentage. Pearson’s Chi-square tests were used to determine any statistical difference about proportions. Continuous variables were compared using independent t-test or Mann–Whitney *U* test. We used the logistic regression model to estimate the impact of preoperative clinical factors on the surgical outcome. The final model was adjusted on confounding factors. A *p* value less than 0.05 was considered statistically significant.

## Results

### Baseline Characteristics

There were 112 patients enrolled in the study, including 69 male and 43 female. The mean age at diagnosis was 55.75 ± 13.19 years (range: 39–83 years). There were 49 patients suffering from large ICH in group A and 63 patients in group B. There were no significant differences in preoperative GCS scores (*p* = 0.142), ICH volume (*p* = 0.153), midline shift (*p* = 0.161), preoperative antiplatelet therapy (*p* = 0.683), time from onset to surgery (*p* = 0.133), and pupillary abnormalities (*p* = 0.126) in both groups. No patients in group A underwent second DC after surgery (Table 1).

**Table 1:** General data of the 112 patients with large putaminal ICH

Variable	Group A (non-DC) *n* = 49	Group B (DC) *n* = 63	*p* Value
Mean age, years^a^	59.63 ± 15.56	53.64 ± 11.75	0.457
Male, no. (%)	31(63.27)	38(60.31)	0.750
Pre-ICH volume (ml)^a^	64.53 ± 15.48	66.99 ± 5.13	0.153
Side of hematoma, no. (%)			0.350
Left	16(32.65)	26(41.27)	
Right	33(67.35)	37(58.73)	
Pre-GCS^b^	6(5,7)	5(4,6)	0.142
Pre-midline shift, mm^a^	11.42 ± 0.29	13.05 ± 0.36	0.161
Preoperative AP, no. (%)	5(10.21)	8(12.70)	0.683
Intraventricular extension, no. (%)			0.589
Present	32(65.31)	38(60.32)	
Absent	17(34.69)	25(39.68)	
Signs of cerebral herniation, no. (%)	37(75.51)	39(61.90)	0.126
Time from onset to surgery (h)^a^	4.82 ± 2.25	4.23 ± 1.31	0.133

*AP* Antiplateleted patients, *DC* decompressive craniectomy, *GCS* Glasgow Coma Scale,* ICH* intracerebral hemorrhage

^a^Values are expressed as the mean ± SD

^b^Values are expressed as median (quartile)

### Evaluation of Efficacy

**Fig. 2:**
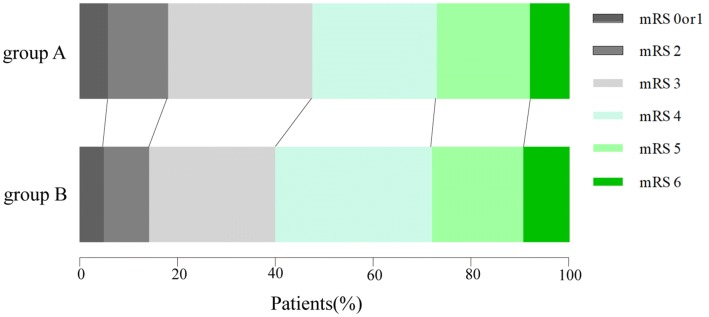
Evaluation of efficacy via comparison of the mRS scores at 3 month between the two groups. Patients in group A (endoscopic hematoma evacuation) received the similar outcome compared to those in group B (DC with hematoma evacuation)

The average rate of hematoma evacuation in groups A and B was 94.48% and 93.80%, respectively. There was no statistical significance between these two groups (*p* = 0.725). There was no difference in the postoperative brain edema volume between the two groups (*p* = 0.382). After 3 months’ follow-up, the mRS scores were 3.44 in group A and 3.64 in group B, with no statistical significance (*p* = 0.709) (Table 2 and Fig. 2).

**Table 2:** Comparison of effective and safe results between two groups in patients with large putaminal ICH

Variable	Group A (non-DC)	Group B (DC)	*p* Value
Hematoma evacuation rate (%)	94.48 ± 0.05	93.80 ± 0.05	0.725
Midline shift reduction(cm)^a^	0.76 ± 0.32	0.80 ± 0.22	0.756
Brain edema(ml)^a^	17.83 ± 5.12	20.88 ± 9.71	0.383
Mortality rate, no. (%)	8.16	9.52	0.538
Postoperative complication, no. (%)			**0.024**
Hydrocephalus	2(4.08)	3(4.76)	
Cerebral infarction	1(2.04)	2(3.17)	
Meningitis	2(4.08)	6(9.52)	
New brain hemorrhage	1(2.04)	3(4.76)	
Severe extra-axial fluid collection	− 0(0)	5(7.94)	
ICP (mmHg)^a^			
ICP (at the end of surgery)	9.00 ± 1.32	7.82 ± 1.83	0.112
ICP (Postoperative, at 1 wk)	10.22 ± 1.22	9.09 ± 1.70	0.092
Pro-mRS(3 month)^a^	3.44 ± 1.33	3.64 ± 1.12	0.709

*DC* decompressive craniectomy, *ICH* intracerebral hemorrhage, *ICP* intracranial pressure, *mRS* modified Rankin Scale

^a^Values are expressed as the mean ± SD; bold type indicates statistical significance

### Evaluation of Safety

**Fig. 3:**
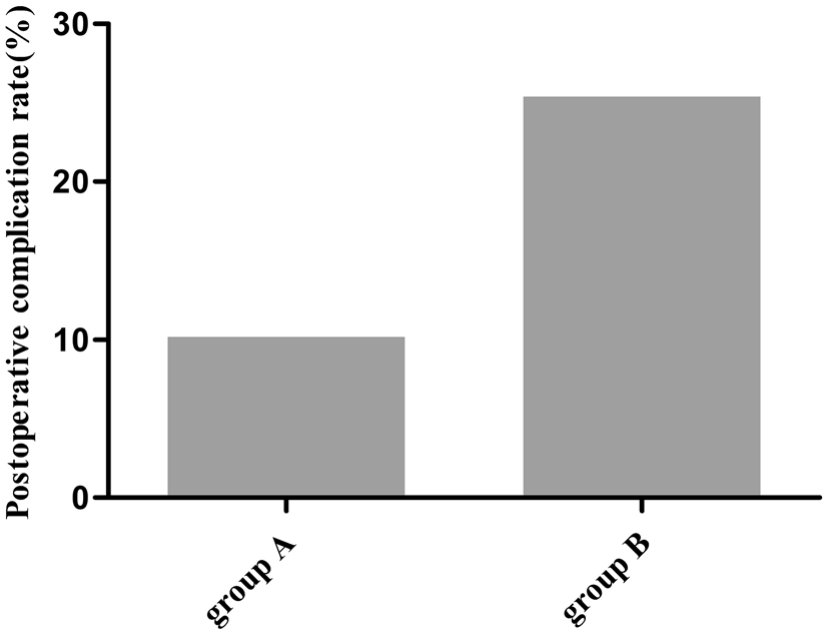
Evaluation of safety via comparison of the postoperative complication rates between the two groups. Group A (endoscopic hematoma evacuation) has a much lower complication rates than that of group B (DC with hematoma evacuation)

The mortality rate in groups A and B was 8.16% (4/49) and 9.52% (6/63), respectively, with no statistical significance between these two groups (*p* = 0.538). Six patients experienced postoperative complications in group A with one suffered from new brain hemorrhage 1 day after surgery, two from hydrocephalus, one from cerebral infarction, and two from meningitis, whereas 19 patients had postoperative complications in group B with three suffered from new brain hemorrhage after surgery, three from hydrocephalus, two from cerebral infarction, six from meningitis, and five from serious extra-axial fluid collection. The postoperative complications showed a significant difference between the two groups (*p* = 0.024) (Table 2 and Fig. 3).

### Factors Associated with Clinical Outcome

The clinical outcome was dichotomized into favorable (mRS 0–3) and unfavorable (mRS 4–6). Univariate logistic regression analyses of the preoperative clinical variables showed that smaller preoperative midline shift (*p* = 0.005) and absent intraventricular extension (*p* = 0.034) have significantly contributed to better outcome. However, age *p* = 0.272), sex (0.691), sides (*p* = 0.583), and pre-GCS (*p* = 0.147) were not correlated with postoperative outcomes. Multivariate logistic regression analyses of the preoperative clinical variables also showed that smaller preoperative midline shift (OR, 2.24; 95% CI, 1.04–5.28; *p* = 0.044) and absence of intraventricular extension (OR, 3.08; 95% CI, 1.33–1.70; *p* = 0.008) were associated with a favorable clinical outcome.

## Discussion

In our present study, we investigated the effectiveness and safety of endoscopic surgery without DC for surgical treatment of large putaminal ICH. We found that there was no difference in the mortality rate and the mRS score comparing the endoscopic hematoma evacuation (group A) with the DC hematoma evacuation (group B). The rate of complications was lower in endoscopic hematoma evacuation (group A) than that of the DC hematoma evacuation (group B). Smaller preoperative midline shift and absent intraventricular extension have significantly contributed to better clinical outcome. This study showed that endoscopic hematoma evacuation achieved similar clinical effect as DC hematoma evacuation.

Surgical evacuation of the intracerebral hematoma can reduce the hematoma mass, decrease ICP, improve regional blood flow, and restrict the release of toxic breakdown products by blood clot. Endoscopic surgery has been gaining popularity as a method for surgical intervention in ICH patients [15–17]. During the surgery, a transparent tube was inserted into the hematoma center, hematoma was evacuated by suction through the working space, and as the sheath was gradually withdrawn, the residual hematoma was removed. Sometimes, residual hematoma could be found by gently adjusting the sheath [18, 19]. Moreover, due to the high pressure nature of the hematoma, its evacuation before DC was easier than after. When an intraoperative hemorrhage occurs, irrigation, compression, or coagulation were needed to secure hemostasis [20–22]. Comparing to craniotomy, endoscopic hematoma evacuation provides a high hematoma evacuation rate with minimal damage to normal brain tissue and lower rebleeding rate [19]. In accordance with previous study, our study also demonstrated a high hematoma evacuation rate by applying endoscopic hematoma evacuation for large ICH with no patients experienced postoperative bleeding.

The debate about surgical removal of large intracerebral hematoma has always focused on either hematoma evacuation or DC. The literature review about large ICH surgery is summarized in Table 3. Brain edema usually occurs after ICH evacuation, especially when the intracerebral hematomas were large [23]. Brain injury, associated brain edema, residual hematoma, and rebleeding resulted in elevation of ICP and finally lead to unsatisfactory outcome. DC can improve cerebral compliance, cerebral oxygen supply [24], and cerebral blood perfusion. As a result, it can reduce the mass effect arising from brain edema, residual hematoma, and rebleeding during the evacuation of ICH [25]. Therefore, some surgeons believe that it is reasonable to choose the DC procedure. However, efficient completion of hematoma evacuation without DC could also reduce the ICP and successfully manage ICP after surgery [6, 26]. In our study, patients with endoscopic hematoma evacuation received similar mortality rate and favorite outcome compared to those with both hematoma evacuation and DC. DC may be unnecessary to treat patients with large ICH, whereas endoscopic hematoma evacuation may present itself several advantages over DC as indicated below. First, all patients were operated via endoscopic surgery within 24 h of hematoma onset. The brain tissue became slack and even collapsed immediately after hematoma evacuation. Brain edema in the surgical area was acceptable. Second, residual hematoma and rebleeding were not developed with properly adjusted sheath and meticulous hemostasis. Third, by releasing CSF through the lumbar catheter drainage and dehydration treatment for postoperative brain edema, the ICP was maintained in the normal range.

**Table 3:** Summary of the literature on surgical treatment of large ICH

Authors and years	Study design	Surgical intervene	No. of pts	Outcome
Murthy et al. (2005)	Retrospective	ICH evacuation with DC	12	The mortality rate was 8.3% and 54.5% had good functional outcome
Takeuchi et al. (2013)	Retrospective	ICH evacuation with DC	21	The mortality rate was 10% and 28.6% of patients with good function
Zhang et al. (2014)	Retrospective	Microscopic ICH evacuation with DC and CSF drainage	33	The mortality rate was 24.3% and 15.1% of patients with good function.
YamaShiro et al. (2015)	Retrospective	Endoscopic ICH evacuation without DC versus microscopic ICH evacuation with DC	43	Endoscope without DC has similar effect in terms of mortality and management of ICP as compared with craniotomy
Hadjiathanasiou et al. (2017)	Retrospective	DC with ICH evacuation versus DC without ICH evacuation	44	Additional ICH evacuation does not seem to be beneficial
Moussa et al. (2017)	Prospective	ICH evacuation with DC versus ICH evacuation	40	DC with hematoma evacuation improved the outcome
Present study	Prospective	Endoscopic ICH evacuation without DC versus DC with endoscopic ICH evacuation	112	Pure endoscope without DC has similar effect in terms of mortality and favor clinical outcome as compared with craniotomy

*CSF* cerebrospinal fluid, *DC* decompressive craniectomy, *ICH* intracerebral hemorrhage, *ICP* intracranial pressure

CSF drainage could increase the likelihood of satisfactory outcomes after surgery. In our procedure, a cannula was placed in the contralateral lateral ventricle during surgery. The main reasons for cannula placement were to reduce ventricular blood and rapidly decompress the brainstem. The cannula was kept open to allow postoperative drainage until no further blood in the third and the fourth ventricles on CT, and then lumbar drain was placed to promote early washout of blood in the subarachnoid space, avoiding irreversible damage to the arachnoid granulations. The main risks involved in the drainage protocols included difficulty during intraoperative ventricular puncture and postoperative intracranial infection. However, puncture based on CT measurements and strict aseptic technique could minimize these risks.

Several delayed complications of DC, including sinking flap syndrome, extra-axial fluid collection, and the development of subdural hematoma, have been reported [27–30]. In addition, hydrocephalus might present in ICH patients treating with DC and hematoma evacuation. The reasons behind this included intraventricular extension and meningitis [31]. This study confirmed that patients operated by DC might get the high incidence of complications, including hydrocephalus. Although patients complicated with hydrocephalus did not need surgical intervention for mild ventriculomegaly and early CSF drainage, they still would undergo long-term hospitalization and slow recovery.

The major strength of this study is to provide an alternative surgical method to neurosurgeons. Patients with large putaminal ICH may not need to experience cranioplasty and thus avoided second operation and extra economic burden. The present study has some limitations. First, despite the lack of significant differences in ICH size, intraventricular hematomas (IVH) size, GCS, and age, these factors may have some influences on the findings because of the small size of the population studied. Second, this is a retrospective analysis without taking microsurgery in comparison. In addition, randomization was not stratified in this study when we divided patient into different groups.

## Conclusions

Our findings indicate that endoscopic hematoma evacuation without DC is safe and effective for large putaminal intracerebral hematoma patients and DC may be unnecessary for rescuing these patients. Multicenter, prospective, randomized, controlled clinical trials are warranted to support our results.
